# The association between dietary pattern and coronary artery disease: A case-control study

**DOI:** 10.34172/jcvtr.2020.48

**Published:** 2020-11-28

**Authors:** Esmaeel Gholizadeh, Parvin Ayremlou, Sakineh Nouri Saeidlou

**Affiliations:** ^1^Food and Beverages Safety Research Center, Urmia University of Medical Sciences, Urmia, Iran; ^2^Clinical Research Development Unit of Imam Khomeini Hospital, Urmia University of Medical Sciences, Urmia, Iran

**Keywords:** Dietary Pattern, Coronary Artery Disease, Cardiovascular Diseases, Principal Component Analysis

## Abstract

***Introduction:*** Dietary patterns are an important factors in the progress of cardiovascular disease. This study aimed to assess the association between dietary patterns and coronary artery disease (CAD).

***Methods:*** A case-control study was carried on 550 participants. Food expenditure was collected using a validated 168-item food-frequency questionnaire. Dietary patterns were extracted by principal component analysis (PCA). Multiple logistic regressions was used to assess the association between dietary patterns and the risk of CAD.

***Results:*** Three major dietary patterns were identified: the "Quasi-Western Pattern" was characterized by higher intakes of sweets and desserts, snacks, legumes, honey or jam, ketchup, mayonnaise, yellow vegetables, potatoes, red meat, refined grains; the "Sugar and Fast foods Pattern" was characterized by higher intakes of sugar, soft drinks, fast foods, high-fat dairy, hydrogenated fats, and the "Quasi-Mediterranean Pattern" was characterized by higher intakes of fruits, cruciferous vegetables, green leafy vegetables, other vegetables, nuts, coffee. In both sexes, the "Quasi-Western Pattern" and the "Sugar and Fast foods Pattern" were positively associated with the risk of CAD. For "Quasi-Western Pattern", adjusted-ORs were (OR: 1.35, 95% CI: 0.99-1.83, *P *= 0.05) and (OR: 1.38, 95% CI: 1.03-1.83, *P *= 0.03)for men and women respectively. The ORs were for "Sugar and Fast foods Pattern" (OR: 3.64, 95% CI:2.25-5.89, P < 0.001) and (OR: 3.91, 95% CI: 2.42-6.63, P < 0.001) for men and women respectively.There was a significant inverse relationship among "Quasi-Mediterranean pattern" and CAD in the crude model in women (OR: 0.7, 95% CI: 0.55-0.89, *P *= 0.0.004).

***Conclusion:*** High adherence to the "Quasi-Western Pattern" and "Sugar-Fast foods Pattern" dietary patterns were associated with a higher risk of CAD. The "Quasi-Mediterranean pattern" reduced the risk of CAD.

## Introduction

Cardiovascular diseases (CVDs) occur due to heart and blood vessel disorders. The different types of CVDs are coronary heart disease, rheumatic heart disease, and other conditions. The presence of atherosclerosis in the epicardial coronary arteries is the characteristics of coronary artery disease (CAD).^1^ An estimated 32·2 million of Non-Communicable Diseases (NCDs) deaths (80%) was due to cancers, cardiovascular diseases, diabetes, chronic respiratory diseases, and another 8·3 million (20%) were from other NCDs.^2^ Annually, 17.9 million people lose their lives due to CVDs. It is estimated that 7.4 million of CVDs deaths were due to Coronary heart disease (CHD) and 6.7 million to stroke.^3^ In the Eastern Mediterranean Region (EMR), 54% of deaths from NCDs are due to CVDs.^4,5^ In Iran, like many developing countries the incidence of CVD has increased and CVDs are the first cause of death, responsible for 46% of deaths ^6^.

The most important CVDs risk factors are unhealthy lifestyle factors. These factors are poor dietary habits, physical inactivity, tobacco use, alcohol harmful using, overweight, and obesity and blood lipids disorders.^7-13^ Poor dietary patterns are important causes of many diseases such as CVDs worldwide.^14,15^ Lack of attention to the nutritional recommendations and choice of foods high in solid fats, added sugars, and processed foods are increasing globally.^16-21^ Different studies have shown that an unhealthy dietary pattern associated with CVDs.^22-25^ Epidemiological and randomized clinical trials (RCTs) studies have shown that an effective factor in reducing of CVDs is changes in lifestyle and having a healthy dietary pattern.^23,26-31^ Therefore, the assessing of dietary patterns has provided a better information for evaluating the association between dietary intake and the risk of diseases than using single food consumption.^17,32^ assessing of dietary pattern is a complex process, it is necessary that employ the methods that evaluate the total dietary intakes instead of individual dietary components.^16^ As well as changes in lifestyle behaviors including avoiding tobacco, being physically active, healthy eating, and balanced dietary pattern, are necessary.^33^ Dietary patterns can show the actual dietary behavior in the population and thus they provide more comprehensive findings.^17,34^

Thus, the importance of healthy eating is widely known, but many persons continue to poor dietary choices. Factor analysis is a useful method for the extraction of dietary patterns based on a correlation matrix of food intakes. Therefore, the current study has investigated the association between dietary patterns and the risk of CAD.

## Materials and Methods

### Study participants

This case-control study was conducted on new cases of CAD. 180 cases and 370 controls were included. In this study, the sampling method was available sampling. The study population was adults (men and women) aged 18-65 years who referred to the Imam Reza hospital (referral hospital) in the Amol city of north-Iran. Participants were recruited from the cardiology department between June 2016 and September 2016. New cases referring to elective coronary angiography with clinical suspicion of CAD were enrolled. The cases were the participants who had been diagnosed based on angiography, stress echocardiogram, nuclear medicine perfusion.

Test, complete lipid profile, C-reactive protein (CRP), and calcium score screening heart scan.

The control group was composed of healthy subjects without any diseases. The exclusion criteria were congenital heart disease, taking oral contraceptives in women, hypertension, hyperlipidemia, diabetes, pregnancy, and lactation.

According to of Odds ratio (OR) equal to 0.53 in a previous study ^35^ and the 37% of deaths due to CVD (P_1_) reported by WHO ^4^, with the 95% CI ($Z_{1-\frac{\dot{a}}{2}}=1.96$) and power of 80% (Z_á-_ = 0.84), the sample size was calculated using the following formulas:

(1) $$P_{2}=\frac{P_{1}\times OR}{1+P_{1}(OR-1)}$$

(2) $$P=\frac{P_{1}+P_{2}}{2}$$

(3) $$C=\frac{Control}{case}=\frac{2}{1}$$

(4) $$n=\frac{\left(1+\frac{1}{C}\right)\times {\left(Z_{1-\frac{\dot{a}}{2}}+Z_{1-\hat{a}}\right)}^{2}\times P(1-P)}{{\left(P_{1}-P_{2}\right)}^{2}}$$

Then, based on the above formulas and 20% of the additional samples, finally, 180 cases and 370 controls were included in this study.

### Dietary intake Assessment

A validated semi-quantitative 168-item food-frequency questionnaire (FFQ) was used for the collection of dietary information.^34,36^ Participants were asked about the frequency of consumption (daily, weekly, monthly, etc) over the past year of each food item .The frequency classification of each food was as follows: occasionally or never , 1–3 numbers /month, 1–2 numbers /week, 3–4 numbers /week, 5–6 numbers /week, 1 number /day, 2 numbers /day, and 3 numbers /day. Then, the frequency classification of each food item was converted to daily intake . Daily intake of food items (gram/day) was calculated based on the reference book “Guides of Coefficients and Household Scales”.^37^ Therefore, the amount of daily intake (gram/day) for food items was calculated by multiplying the portion sizes to the consumption frequency. For the consumed foods items as weekly or monthly, the product of multiplication (portion size by consumption frequency) divided to the seven or thirty, respectively. Also, in addition to the above calculations, the number of months for the seasonal fruits were multiplied and divided to 365 days. Then, the FFQ food items were classified into 34 separate food groups based on the similarity in their nutrient profile or previous studies ^24,29,38^ (Table 1). If the food item was very different from other items (e.g. eggs, tea, and coffee), it was classified independently as a food group. Finally, factor analysis was used to identify the major dietary patterns.

**Table 1:** Food groups used in the factor analysis

**Food items**	**Subgroups**
1- Refined grains	Lavash bread(Iranian bread), Baguette, Rice, Macaroni
2- Whole grains	Iranian dark Bread (Barbari, Sangak, Taftoon), Barley, Bulgur
3- High-fat dairy	High-fat milk, high-fat yogurt, cream cheese, cream and butterfat, ice cream
4- Low-fat dairy	Low-fat milk, Low-fat yogurt, White cheese, curd, Dough
5- Visceral Meat	Heart, liver, tongue, brain, chest and pancreas and abdomen
6- Red Meat	Beef, lamb, minced meat
7- Poultry	Chicken
8- Egg	Egg
9- Cruciferous vegetables	Cauliflower, cabbage
10- Yellow vegetables	Carrots
11- Green leafy vegetables	Spinach, Lettuce
12- Other vegetables	Tomatoes, Cucumber, Eggplant, Onion, Green Pea and Beans, Pumpkin, Mushrooms, red and green Peppers, turnip, Corn and maize, Garlic
13- Potatoes	Boiled and fried potatoes
14- Fruits	Pears, apricots, cherries, apples, raisins or grapes, bananas, cantaloupe, watermelon, oranges, grapefruit, kiwi, strawberries, peaches, nectarine, tangerine, mulberry, plums, persimmons, pomegranates, lemons, pineapples, fresh figs and dates
15- Legumes	Chickpeas, lentils, beans, peas, soybeans
16- Non-hydrogenated fats	Vegetable oils (except for olive oil)
17- Hydrogenated fats	All types of Solid oil, Animal oil, Animal butter, Margarine
18- Olive	Olives, olive oil
19- Nuts	Almonds, Peanuts, Walnuts, Pistachio, Hazelnuts, Seeds
20- Salt	Salt
21- Fast foods	Pizza, Sandwich, Sausage, Hamburger
22- Snacks	Puff, Chips
23- Sweets and desserts	All types of Sweets, Chocolates, Cakes, and muffins
24- Sugars	Sugars, chocolates, candies, Gaz (an Iranian confectionery made of sugar, nuts, and tamarisk), Sohan
25- Tea	Tea
26- Coffee	Coffee, Nescafe
27- Honey or jam	honey or jams
28- Ketchup	Ketchup
29- Mayonnaise	Mayonnaise
30- Fishes	Any fish
31- Soft drinks	Soft drinks
32- The Broth	Broth
33- Pickle	Any Pickle
34- Tuna	All types of Canned fish

### Other Study measurements

The demographic characteristics including age, sex, education level,, and marital status family history of CVD, and family history of hypertension were obtained via face to face interviews with participants. The body weight was measured in light clothing and without shoes to the nearest 0.01 kg using a digital scale. Height was measured without shoes in a standing position, using a tape measure with an accuracy of 0.1 cm Body mass index (BMI) was calculated by dividing weight (kg)/height (m^2^). Waist circumference (WC) was measured using the tape measure in standing with feet shoulder-width apart position in the area between the hip bone and under the navel.

### Statistical analysis

Continuous normal variables were presented with means±SD and values for categorical variables were described as the number of cases and percentages. The baseline characteristics were compared between cases and controls using the Person’s Chi-square Test and the mean of continuous variables were compared between two groups by independent T-test. The normality of data was examined using the Kolmogorov-Smirnov test.Principal component analysis (PCA) was conducted to identify the major dietary patterns based on the 34 food groups (Table 1). Components were rotated by varimax rotation with Kaiser Normalization. The numbers of components retained were specified using a combination of the eigenvalues (> 1.5), in the scree plot. For each participant, factor scores of dietary patterns (DPs) were calculated observed intakes of the ingredient food items weighted by factor loadings. Factor loadings >|0.20| were considered to contribute to the component and were used to name the dietary patterns. ORs and their 95% confidence intervals (CI) were computed for the assessing of association between dietary patterns scores and the risk of CAD. The ORs calculated by logistic regression in crude and two adjusted models. In all regression models for each dietary pattern, model 1 was adjusted for age (continues), marital status, and education level, family history of CAD and family history of hypertension (HTN) (as categorical), in model 2 in addition to model 1, waist circumference, hip circumference (HC), waist to hip ratio (WHR) and the BMI, as covariates were entered to the model. To control for confounder effect of sex, the stratified analysis performed by sex and other variables adjusted as covariates in regression models. All statistical analyses were carried out using the Stata software version 14.0 (College Station, Texas 77845 USA) and the* P* value>0.05 was considered statistically significant.

## Results

Table 2 shows the main baseline characteristics of participants (180 cases and 370 controls). The sex, age groups, marital status, and education level were statistically significant between cases and controls. In cases, females (68.9%) and the age group >50 years (63.9%) had the highest frequency. Married participants were more than another marital status in both groups. The CAD risk was lower in participants with higher education levels. The mean of all anthropometric indices was higher in cases compared to controls and these differences were significant between two groups (*P* < 0.05).

**Table 2:** Comparison of the baseline characteristics between cases and controls

**Variables**		**Case (n=180)**	**Control (n=370)**	***P*** ** value**_ Trend_
Sex, n (%)	Male	56 (31.1)	203 (54.9)	<0.001^¶^
	Female	124 (68.9)	167 (45.1)	
Age (year), n (%)	<40	27 (15)	177 (47.8)	<0.001^¶^
	50-40	38 (21.1)	85 (23)	
	>50	115 (63.9)	108 (29.2)	
Marital status, n (%)	Single	5 (2.8)	90 (24.3)	<0.001^¶^
	Married	162 (90)	277 (74.9)	
	Divorced	13 ( 7.2)	3 (0.8)	
Education Level, n (%)	Elementary	105 (58.3)	100 (27)	<0.001^¶^
	Middle school	30 (16.7)	60 (16.2)	
	High school	35 (19.4)	132 (35.7)	
	Academic	10 (5.6)	78 (21.1)	
Family history of CVD^*^	Yes	44 (24.4)	91 (24.6)	0.96^¶^
	No	136 (75.6)	279 (75.4)	
Family history of hypertension	Yes	97 (53.9)	159 (43)	0.02^¶^
	No	83 (46.1)	211 (57)	
BMI(Kg/M^2^), mean±SD	-	32.27±5.4	26.62±4.5	<0.001^¥^
Weight, mean±SD	-	80.29±14.4	72.1±14.8	<0.001^¥^
Waist circumference	-	93.95±10.1	83.26±10.8	<0.001^¥^
Hip circumference	-	107.2±10.6	99.1±8.2	<0.001^¥^
WHR	-	0.88±0.07	0.84±0.09	<0.001^¥^

Abbreviations: BMI, Body Mass Index; WHR, Waist to the Hip circumference ^*^Cardiovascular Disease ^¶^Analyzed by Pearson chi-square test ^¥^Analyzed by two-sample Independent *T* test

**Figure 1:**
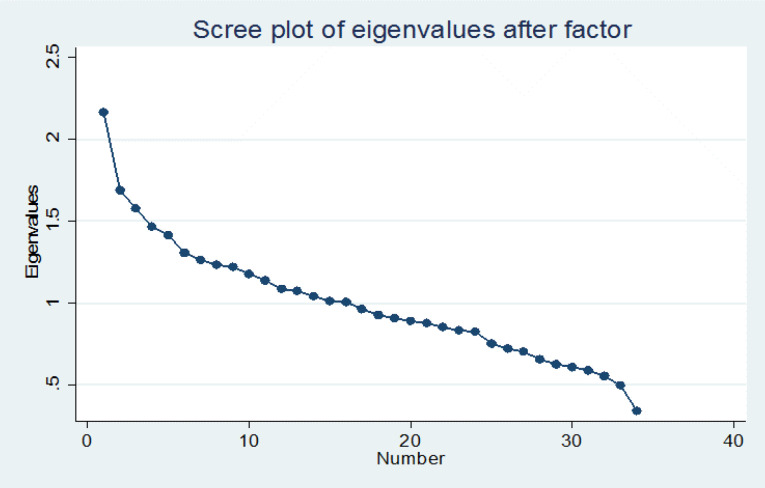
Scree plot by principal component analysis

Three factors were considered as major dietary patterns and were tagged based on our interpretation of the data (Figure 1). These factors explained approximately 16% of the total variance. Table 3 shows the factor loading of major dietary patterns by PCA analysis. Positive loadings demonstrate a positive association with the pattern, while negative loadings demonstrate an inverse association with it. The PCA identified three major dietary patterns in all participants as follows. These three distinct dietary patterns based on our interpretation of the data and similar to extracted patterns in previous studies, labeled as “Quasi-Western”, “Sugar and Fast foods” and “Quasi-Mediterranean” dietary patterns. The western dietary pattern usually was specified by a higher intake of processed meat,red meat, refined grains, dessert and sweets , French fries, and high-fat dairy products.^39^ In the current study, the “Quasi-Western” pattern had a high amount of sweets and desserts, snacks, legumes, honey or jam, ketchup, mayonnaise, yellow vegetables, potatoes, red meat, refined grains, and was explained responsible for 5.8% of the total variance. The second pattern similar to previous studies ^40,41^ and because the high factor loading of sugar and fast foods were named “Sugar and Fast foods”. In this study, the “Sugar and Fast foods” pattern which loaded heavily on sugar, soft drinks, fast foods, high-fat dairy, hydrogenated fats, and it was explained 5.3% of the variance. The main ingredients of the Mediterranean diet include daily ingredients of fruits, vegetables, healthy fats and whole grains, . Weekly intake of poultry, fish,eggs and beans. Moderate portions of dairy products.^42^ In this study the “Quasi-Mediterranean” pattern was identified as the high amount of fruits, cruciferous vegetables, green leafy vegetables, other vegetables, nuts, coffee, and it was explained 4.9% of the variance. These three patterns were explained by approximately 16% of the total variance. Sampling adequacy and inter-correlation of factors were supported by Kaiser-Meyer-Olkin measure (KMO value=0.523) and Bartlett’s test of Sphericity <0.001, respectively. The ORs and their 95% CI for CAD by scores of the three dietary patterns stratified by sex are shown in Table 4. “Quasi-Western Pattern “in both sexes, was positively associated with the risk of CAD. As the OR in the crude model was (OR: 1.29, 95% CI: 0.98-1.71, *P* = 0.07) in men and (OR: 1.27, 95% CI: 1.001-1.61, *P* = 0.04) in women. In model 1 the ORs were (OR: 1.35, 95% CI: 0.99-1.83, *P* = 0.05) and (OR: 1.38, 95% CI: 1.03-1.83, *P* = 0.03) for men and women respectively and this differences were significant in both models for women and model 1 for men (as borderline). In the last adjusted model, with the increase of one unit in the score of “Quasi-Western Pattern”, the risk of CAD increases by 31% in men.

**Table 3:** Factor loading^a^ of Major dietary patterns in all participants

	**Dietary patterns**		
**Food groups**	**Quasi-Western**	**Sugar and fast foods**	**Quasi-Mediterranean**
Sweets and desserts	0.687	-^b^	-0.236
Snacks	0.642	-	-0.429
Legumes	0.459	-	0.284
Honey or jam	0.221	-	-
Ketchup	0.257	-	-
Mayonnaise	0.203	-	-
Yellow vegetables	0.333	-	-
Potatoes	0.269	-	-
Fruits	-	-	0.303
Cruciferous vegetables	-	-	0.563
Green leafy vegetables	-	-	0.614
Other vegetables	-	-	0.421
Refined grains	0.202	-	-
Whole grains	-	-	-
Sugars	-	0.570	-
Soft drinks	0.228	0.505	
Fast foods	-	0.433	-
High-fat dairy	-	0.415	-
Low-fat dairy	0.209	-0.417	-
Hydrogenated fats	-	0.371	-
Organ Meat	-	-	-
Red Meat	0.264	-	-
Poultry	-	-	-
Egg	-	-	-
Non-hydrogenated fats	-	-	-
Olive	-	-	-
Nuts	-	-	0.269
Salt	-	0.283	-
Tea	-	-	-
Coffee	-	-	0.205
Fish	-	-	-
Broth	-	0.371	-
Pickle	-	-	-
Tuna	-	-	-
Eigenvalue	2.164	1.69	1.578
% of variance explained	5.764	5.264	4.95
% of cumulative variance explained	5.764	11.028	15.978

^a^Extraction Method:Principal Component Analysis, Rotation Method: Varimax with Kaiser Normalization. ^b^blank represent Factor loadings < l0.2l for simplicity. Kaiser-Meyer-Olkin measure of sampling adequacy: 0.523

**Table 4:** The Odds Ratio (OR) and 95% CI for the association of coronary artery disease with scores of dietary patterns

		**Men(n=259)**		**Women(n=291)**	
**Dietary pattern**		**OR (95% CI)**	***P*** ** value**	**OR (95% CI)**	***P*** ** value**
Quasi-Western	Crude model	1.29 (0.98-1.71)	0.07	1.27 (1.001-1.61)	0.04
	Model 1	1.35 (0.99-1.83)	0.05	1.38 (1.03-1.83)	0.03
	Model 2	1.31 (0.93-1.83)	0.12	1.09 (0.78-1.54)	0.61
Sugar and fast foods	Crude model	3.45 (2.34-5.09)	<0.001	3.86 (2.7-5.51)	<0.001
	Model 1	3.28 (2.15-5.01)	<0.001	3.68 (2.45-5.52)	<0.001
	Model 2	3.64 (2.25-5.89)	<0.001	3.91 (2.42-6.33)	<0.001
Quasi-Mediterranean	Crude model	0.9 (0.66-1.23)	0.51	0.7 (0.55-0.89)	0.004
	Model 1	0.97 (0.69-1.38)	0.88	0.77 (0.58-1.03)	0.07
	Model 2	0.95 (0.65-1.39)	0.79	0.84 (0.61-1.17)	0.31

Model 1 is adjusted for Age (continues), Marital status and education level, family history of CAD, and family history of HTN (as categorical). Model 2 is adjusted for Model 1 covariate plus BMI, Waist circumference, HC, WHR (as continues) Abbreviations: BMI (Body Mass Index), WHR (Waist to hip ratio), HC (Hip circumference), CAD (Coronary Artery Disease), HTN (Hypertension)

In crude and two adjusted models, “Sugar and Fast foods Pattern” had a significant inverse association with the risk of CAD in both sexes. Therefore, the ORs were in the last model (OR: 3.64, 95% CI: 2.25-5.89, *P* < 0.001) and (OR: 3.91, 95% CI: 2.42-6.63, *P* < 0.001) for men and women respectively.

The “Quasi-Mediterranean pattern” reduces the risk of CAD, although this difference was not statistically significant in adjusted last model in both sexes (OR: 0.95, 95% CI: 0.65-1.39, *P* = 0.79 in men and OR: 0.84, 95% CI: 0.61-1.17, *P* = 0.31 in women). but there was a significant inverse relationship among“Quasi-Mediterranean pattern” and CAD in the crude model in women (OR: 0.7, 95% CI: 0.55-0.89, *P* = 0.0.004).

## Discussion

In this study, there was a positive significant association between “Quasi-Western pattern” and “Sugar-Fast foods pattern” and the risk of CAD. Our findings showed that the “Quasi-Western pattern” and the “Sugar-Fast foods pattern” increased the risk of CAD in both men and women. While adherence to the “Quasi-Mediterranean pattern” decreased the CAD incident. Although the diet of each population has specific characteristics the bulk of dietary patterns share a high expenditure of proteins from plant sources (legumes and nuts), fruit, vegetables , a high intake in fats, meat and proceed foods. The dietary patterns that we derived from the PCA are similar to the extracted patterns in other previous studies.^22,38,43,44^ The variance mentioned by the three dietary patterns (Quasi-Western: 5.8%, Quasi-Mediterranean 5.3%, and Sugar- Fast foods: 4.9%) is similar to the dietary patterns insulated by PCA in other studies.^17,38^

The main characteristic of western dietary pattern as the unhealthy diet is a higher intakes of red meat, refined grains, processed meat, French fries, sweets and dessert, and high-fat dairy products. Similar to our findings, several studies throughout the world have shown that the western dietary pattern increases the risk of CVDs. ^7,17,38,43-47^ The western dietary pattern was extracted in Oikonomou`s and et al study, involve higher intakes of fat, red meat, carbohydrates, and minimal expenditure of green leafy vegetables and fruits and was associated positively with the severe of CAD.^43^ Another study showed that higher scores of dietary pattern with high intakes of margarine, meat, sauce, and poultry and low intakes of vegetarian dishes, wine, vegetables, and whole-grain cereals significantly increases the risk of CAD.^48^ Findings of a study by Drake and et al showed that the western dietary pattern was inversely associated with high-density lipoprotein (HDL) and positively with the diastolic and systolic blood pressure, fasting glucose and insulin.^45^ Dyslipidemia including a high level of total cholesterol, triglyceride, low-density lipoprotein (LDL), and low level of HDL are the main risk factors for atherosclerosis and CVD.^7,12^

The current study showed that the “Sugar- Fast foods” dietary pattern was directly associated with the risk of CAD as in the last adjusted model, the OR was 3.91. Sugar-sweetened beverages overconsumption as a poor diet quality was related to increasing in intra-abdominal obesity and ectopic lipid deposition in the liver, cardiometabolic, and also the CVD risk factors ^20,22^. Consistent with our findings, a study by Yang and et al among United States adults had shown that receiving calories from added sugar was positively associated with the risk of CVD mortality. As the hazard ratios (HRs) of CVD mortality in Fifth quintile was 2.43 compared to the 1st quintile and also Adjusted HRs were 1.30 and 2.75 in participants who consumed 10-24.9% and 25.0% or more calories from added sugar comparing those who consumed less than 10.0% of calories from added sugar, respectively.^49^

The main components of the Mediterranean diet include daily consumption of vegetables, fruits, whole grains, and healthy fats. Weekly intake of fish, poultry, beans, and eggs. Moderate portions of dairy products.^44^

This study indicated that the inverse association between the “Quasi-Mediterranean” dietary pattern and the risk of CAD although this difference was not significant. As after adjusting the potential confounders, the OR for the 4^th^ quartile was 0.74 compared to the 1st quartile. Our findings support the role of a Mediterranean dietary pattern in the prevention of CVD.^27,33,48,50-55^

The effective consequence of the Mediterranean dietary pattern on health can explain by decreasing inflammatory markers and endothelial dysfunction.

Mediterranean dietary pattern causes reductions in weight, blood pressure, cholesterol, and LDL and an increase in HDL levels as the main affected factors on CVD.^28,48,51-53,56,57^ Studies showed that the consumption of low intakes of sugar-sweetened beverages, Western fast foods, fat, poultry, and high intakes of vegetables, fruits, processed meat, whole grains, and unsaturated cooking oil significantly reduced the LDL, total cholesterol level, fasting triglyceride, diastolic blood pressure, BMI and WC .^58-60^

The finding of another study showed that higher adherence to a dietary pattern with high fresh fruit, vegetables, and whole grains intakes was inversely related to the risk of CVD.^61^

In summary, unhealthy dietary patterns are directly associated with the risk of CAD. Dietary pattern-based investigations provide important and useful information about the patterns among people. The evidence reviewed shows that it is important to consider total dietary patterns for CVD prevention. Findings of dietary pattern-based researches can use for the reduction of CVD risk factors. Dietary recommendations should be food and dietary pattern-based, not nutrient targets.

This study has several potencies, it is a case-control study with large sample size. The potential covariates were adjusted for confounder variables in regression models. The food consumption was assessed by a validated-FFQ questionnaire and dietary patterns derived from the PCA method. In interpreting the results of the study, we must pay attention to the biases as study limitations. Therefore, the probability of information bias and the recall bias cannot be ignored for case-control studies. Another limitation of this study was available sampling so that the sex, age groups, and physical activity were not matched between two groups but the analysis was performed stratified by sex and adjusted for other variables.

## Conclusion

In conclusion, the “Quasi-Western” and “Sugar-Fast foods” dietary patterns were positively associated with the risk of CAD. An inverse association detected between the “Quasi-Mediterranean” dietary pattern and the risk of CAD although this difference was not significant in the last adjusted models in both sexes.

## Acknowledgments

The authors appreciate statistical counselors of the Clinical Research Development Unit of Imam Khomeini Hospital, Urmia University of Medical Sciences. The authors certify that there are no conflicts of interest.

## Competing interests

The authors declare that they have no conflict of interest.

## Ethical approval

This study was approved by the ethics committee of Urmia University of Medical Sciences with number ID: ir.umsu.rec.1395.69. All participants signed a consent form.

## Funding

The authors declare that they have no funding source.
